# The effect of preoperative stoma site marking on risk of stoma-related complications in patients with intestinal ostomy—protocol of a systematic review and meta-analysis

**DOI:** 10.1186/s13643-021-01684-8

**Published:** 2021-05-12

**Authors:** Charlotte Mareike Kugler, Jessica Breuing, Tanja Rombey, Simone Hess, Peter Ambe, Erich Grohmann, Dawid Pieper

**Affiliations:** 1 Institute for Research in Operative Medicine, Witten/Herdecke University, Ostmerheimer Str. 200, 51109 Cologne, Germany; 2 GFO Kliniken Rhein-Berg, Vinzenz Pallotti Hospital, Bergisch Gladbach, Germany; 3 Department of Health, Witten/Herdecke University, Cologne, Germany; 4 Deutsche ILCO e.V., Bonn, Germany

**Keywords:** Meta-analysis, Systematic review, Ostomy surgery, Stoma site marking

## Abstract

**Background:**

An intestinal ostomy is an artificial bowel opening created on the skin. Procedure-related mortality is extremely rare. However, the presence of an ostomy may be associated with significant morbidity. Complications negatively affect the quality of life of ostomates. Preoperative stoma site marking can reduce stoma-related complications and is recommended by several guidelines. However, there is no consensus on the procedure and recommendations are based on low-quality evidence.

The objective of the systematic review will be to investigate if preoperative stoma site marking compared to no preoperative marking in patients undergoing intestinal stoma surgery reduces or prevents the rate of stoma-related complications.

**Methods:**

We will include (cluster-) randomised controlled trials and cohort studies that involve patients with intestinal ostomies comparing preoperative stoma site marking to no preoperative marking and report at least one patient-relevant outcome. For study identification, we will systematically search MEDLINE/PubMed, EMBASE, CENTRAL and CINHAL as well as Google Scholar, trial registries, conference proceedings and reference lists. Additionally, we will contact experts in the field. Two reviewers will independently perform study selection and data extraction. Outcomes will be prioritised based on findings from telephone interviews with five ostomates and five ostomy and wound nurses prior to conducting the review. Outcomes may include but are not limited to stoma-related complications (infection, parastomal abscess, hernia, mucocutaneous separation, dermatological complications, stoma necrosis, stenosis, retraction and prolapse) or other patient-relevant postoperative endpoints (quality of life, revision rate, dependence on professional care, mortality, length of stay and readmission). We will use the ROBINS-I or the Cochrane risk of bias tool to assess the risk of bias of the included studies. We will perform a meta-analysis and assess the certainty of evidence using the GRADE approach.

**Discussion:**

With the results of the systematic review, we aim to provide information for future clinical guidelines and influence clinical routine with regard to preoperative stoma site marking in patients undergoing ostomy surgery. When the evidence of our systematic review is low, it would still be a useful basis for future clinical trials by identifying data gaps.

**Systematic review registration:**

PROSPERO registration number: CRD42021226647

**Supplementary Information:**

The online version contains supplementary material available at 10.1186/s13643-021-01684-8.

## Background

### Rationale

An ostomy, also called stoma, defines an artificial opening of an internal organ on the surface skin. An intestinal ostomy therefore comprises of an artificial bowel opening created on the skin to evacuate bowel content [1]. Other forms of stomata include tracheostoma and urostoma [2]. However, this systematic review (SR) focuses on intestinal ostomies which can be created using the small bowel (ileum) or the large bowel (colon). Furthermore, ostomies can be created electively or emergently and they can be either temporary or permanent. Temporary ostomies are routinely performed to ameliorate the consequences of anastomotic insufficiency following colorectal surgery [1]. Permanent ostomies are used as a definite solution to an underlying problem which is usually not amendable via other methods. However, there is a considerable risk of temporary ostomies becoming permanent [1, 3]. Another feature of intestinal ostomies is the number of openings on the skin. A terminal ostomy has just one opening because the entire bowel circumference is sutured to the skin following a complete disruption of the bowel continuity. Loop ostomies on the other hand have two openings at the level of the skin. The anterior wall of the bowel is opened and sutured to the skin while the posterior bowel wall remains intact. Loop ostomies are usually used in situations when bowel continuity can be restored [1, 2]. Frequent indications for creating an intestinal ostomy include bowel cancer, bowel ischemia, inflammatory bowel disease, anal incontinence, chronic obstipation or complicated diverticular disease [2].

Statistics on ostomates are relatively rare and vague. In the USA, the number of ostomates is estimated between 750,000 and 1 million [4, 5] which corresponds to a prevalence of 0.2% and over 100,000 new procedures are performed each year [3, 4]. The German health insurer BARMER estimated that the prevalence of ostomates was 0.2% in Germany in 2011, based on patients consuming stoma-related medical aids [6], corresponding to a total of about 160,000 ostomates. These data include carriers of both intestinal and urinary ostomies [6]. According to the Federal Bureau of Statistics of Germany, about 64,601 intestinal ostomy-related procedures were performed in Germany in 2019. This is approximately 79 ostomy-related procedures per 100,000 residents in Germany [7]. This figure has increased by 24% since 2005, when it was 52,035 procedures. The global number of years lives with disability for patients with stoma due to colon and rectum cancers who survived beyond 10 years was 22,100 years (95% confidence interval: 15,300 to 30,000 years) in 2017 [8].

In elective stoma creation, procedure-related mortality is extremely rare. A SR that included 18 randomised controlled trials (RCT) on intestinal ostomy found that no deaths were attributed to creation of the stoma [9]. However, significant morbidity is associated with intestinal ostomy including both early complication, occurring within the first 30 days after stoma creation, and late complication, occurring after 30 days following stoma creation [1, 3]. Such complications may include peristomal conditions, parastomal hernia, stoma prolapse, retraction, stoma stenosis, ischemia and necrosis and high output stoma [1, 3, 9–11]. Some of these complications, e.g. stoma necrosis, prolapse, retraction, stenosis and parastomal hernia, may require surgical correction [1, 3]. In 2019, over 9,656 revision procedures were performed in Germany [7]. Furthermore, stoma-related complications have been shown to negatively affect the quality of life of ostomates [12]. Besides the negative effects of these complications on the ostomates’ perspective (quality of life) [13, 14], the management of stoma-related complications also constitutes a substantial burden to the health care system [6].

Besides the height of the stoma above the skin [15], a relevant predisposition to stoma-related complications is the stoma site [10, 16]. An optimal ostomy position may prevent many stoma-related complications [1]. Based on our clinical experience and limited, retrospective literature, choosing the right site for an ostomy may constitute a major step in reducing and preventing stoma-related complications [17, 18].

The Charter of Ostomates Rights [19], which was issued by the International Ostomy Association Coordination Committee in June 1993 and last revised by the World Council in 2007, states that:

The Ostomate shall […] have a well-constructed stoma placed at an appropriate site, and with full and proper consideration to the comfort of the patient.

The available data suggests that preoperative stoma site marking and education by wound and ostomy therapists significantly reduce early stoma-related complications [17, 18, 20] and anxiety [18] and increase quality of life [20, 21]. On the contrary, a lack of preoperative stoma site marking was found to be a significant risk factor for problematic stomas [10].

Preoperative marking of the potential stoma site is recommended in several guidelines, including the American [22] and German guidelines [23]. However, there is no consensus on how to mark and who does the marking. It is generally accepted that preoperative marking is best performed by the leading surgeon or an experienced member of the surgical team. Another accepted option is to have an ostomy and wound nurse do the marking [23, 24]. Marking should be done during counselling, by either the surgeon or the ostomy nurse, and the potential stoma site should be marked with the patient in different positions: lying, sitting and standing [23, 24]. It is recommendable to mark the bony structures of the abdominal walls as well as visible skin folds. This permits an optimal selection of the future stoma site away from folds and bony abdominal prominents. The patient should be encouraged to play an active part in this process. One could also let the patient try an ostomy bag, so they can find out if the selected position is optimal [24].

To date, there is no high-quality SR investigating the effect of preoperative stoma site marking on stoma-related complications in patients with intestinal ostomy. The SR by Colwell and Gray published in 2007 [25] includes three studies published between 1997 and 2006 [17, 26, 27] with heterogeneous effects of preoperative stoma site marking on different outcomes. The authors conclude that preoperative site marking may reduce the incidence of postoperative complications but the evidence was sparse. This review has certain methodological limitations, for example it lacks a risk of bias (RoB) assessment of the included studies and is probably out of date as it is estimated that half of SRs are out of date after 5.5 years [28]. A recent SR by Hsu et al. in 2020 [29] focusing on intestinal and urinary stoma included 10 studies. This SR is largely limited by its low methodological quality: The authors did not publish a protocol, did not search reference lists nor trial registries and took a long time from search (January 2018) to publication (May 2020). They did not report having performed selection of studies in duplicate, did not use appropriate methods for statistical combination of results nor did they try to explain heterogeneity or evaluate the quality of the body of the evidence. Furthermore, we identified studies which were not included in that review [29]: two studies included by Colwell and Gray [26, 27] and a study published by Cakir and Ozbayir [20] which indicated that the 6-month quality of life score of the patients receiving preoperative stoma marking was higher than that of the control group.

### Objectives

The objective of the SR will be to investigate if preoperative stoma site marking compared to no preoperative marking in patients undergoing intestinal stoma surgery reduces the risk of stoma-related complications. Our aim is to inform future clinical and political decision-making related to preoperative stoma site marking and/or future clinical trials.

## Methods

We adhered to the Preferred Reporting Items for Systematic Reviews and Meta-Analyses Protocols (PRISMA-P) checklist for writing of this protocol (see Additional file 1) [30].

### Eligibility criteria

- Participants: We will include studies on patients with intestinal ostomies. We will make no differences regarding the type of ostomy: ileostomy vs. colostomy, temporary vs. permanent, loop vs terminal. Equally, we will make no restrictions to the indication for ostomy creation, i.e. the underlying diagnosis, or the urgency of ostomy creation, i.e. elective vs. emergency surgery.
- Intervention: The intervention is preoperative stoma site marking. As there is no standardised definition, we will use the definition provided by the primary study authors. There will be no restriction with regard to the person (nurse, physician etc.) who performs preoperative stoma site marking, nor to other actions performed preoperatively.
- Comparator: The comparator is no preoperative stoma site marking. There will be no restrictions concerning other actions performed preoperatively.
- Outcomes: We will include studies reporting data on patient-relevant endpoints. We will determine which endpoints are relevant to patients and prioritise endpoints by conducting semi-structured telephone interviews with patients and ostomy care nurses prior to the conduct of the review. We provide a list of potential outcomes under the “Outcomes and prioritisation” section.
- Design: We will include RCTs, cluster RCTs and cohort studies (observational studies including registry-based studies). However, based on our preliminary searches and considering that it may be considered unethical to randomise patients to preoperative stoma site marking or no preoperative stoma site marking, we do not expect to identify any RCTs or CRCTs that meet our inclusion criteria. Therefore, the best available evidence will most likely consist of cohort studies.

At least 80% of included participants of each study arm must fulfil the inclusion criteria; otherwise, the study will be excluded (e.g. if more than 20% of patients have urostoma instead of intestinal stoma).

### Information sources

We will search the following databases to identify relevant studies:

- MEDLINE (via PubMed): inception to present
- EMBASE (via EMBASE): inception to present
- CENTRAL (via Cochrane Library): inception to present
- CINHAL (via EBSCO): inception to present
- Google Scholar (via Google Scholar)

We will search manually for additional studies by:

- Cross-checking the reference lists of all included primary studies
- Cross-checking the reference lists of relevant SRs

We will search the following trial registries:

- Clinicaltrials.gov
- German Clinical Study Register (DRKS)
- International Clinical Trials Registry Platform (ICTRP)

Furthermore, we will contact experts for additional studies. Experts will be identified by:

- Corresponding authors of all included primary studies
- Corresponding authors of all relevant SRs

We will hand search available abstracts from conference reports of the following conferences:

- Stoma-related skin problems
- Wound and ostomy care
- Quality of life in ostomate
- Stoma complications

### Search strategy

We will conduct a literature search to identify all published and unpublished studies. The research team will develop the search strategy in collaboration with an experienced librarian according to the *Peer Review of Electronic Search Strategies* (PRESS) guideline [31]. We will apply no restrictions regarding language, publication data and publication status. A draft of the PubMed search strategy is presented below:

"Surgical Stomas"[Mesh] OR "Enterostomy"[Mesh] OR stoma*[tiab] OR ostom*[tiab] OR colostom*[tiab] OR ileostom*[tiab] AND (marking[tiab] OR siting[tiab] OR “stoma site”[tiab])

### Data management

We will import all potentially relevant hits into EndNote (Clarivate Analytics, version X9.3.1) and subsequently to Rayyan [32]. Duplicate records will be removed prior to the selection process.

### Selection process

Two reviewers will independently screen titles and abstracts of all unique records using Rayyan [32]. We will retrieve the full texts of all potentially relevant articles. Two reviewers will then independently review full-text articles in detail regarding inclusion criteria. Both reviewers must consider an article eligible for it to be included. Discrepancies will be resolved by discussion; if necessary, a third reviewer will be involved. In case of any uncertainty, we will contact the authors of the primary studies.

### Data collection process

It is important for SRs to make their data available in the long turn. By promoting open science and data sharing, the efficiency in conducting SRs can be expected to be largely increased. At the same time, unnecessary duplication resulting from recurring data extraction of the same primary studies included in multiple SRs can be decreased. Therefore, we will use the updated Systematic Review Data Repository (SRDR+) launched by The Brown University Evidence-based Practice Center (Brown EPC) in the USA. It is free for use and has been funded by The Agency for Healthcare Research and Quality (AHRQ). We will use SRDR+ to organise all data of our SR. In addition, SRDR+ is also intended as a repository for extracted data. Thus, our data will be freely available to other researchers working on similar topic in a long term.

A standardised data abstraction form will be developed and calibrated with the team. Using a random sample of five of the included studies, the data abstraction form will be pilot-tested, and revised, if necessary. Two reviewers will independently perform data extraction of the included studies using the standardised and piloted data abstraction form. Then, both reviewers will check each other’s versions for completeness and accuracy. Any discrepancies will be resolved by discussion, involving a senior researcher if necessary. Discrepancies related to clinical aspects will be discussed with a clinical expert in our research team. We will contact primary study authors via email in case of missing data or uncertainties related to the data (e.g. follow-up time points).

### Data items

We will extract data on the following items:

- Funding
- Sample size (number of included patients)
- Patients eligibility criteria
- Type of hospitals (e.g. teaching hospital)
- Surgeon characteristics (e.g. years of experience, colorectal vs. general surgeon)
- Characteristics of the surgery (e.g. placement of a peristomal mesh)
- Year(s) of data collection
- Follow-up period
- Country/region
- Data source (clinical vs. administrative)
- Database/registry (if any)
- Definition of preoperative stoma site marking
- Procedure characteristics (e.g. types of marker used, type of person who performed marking)
- Patient characteristics (age, sex, diagnosis, comorbidities, BMI)
- Outcomes
- Effect measures (unadjusted and adjusted) with corresponding confidence intervals and/or p-values
- Adjusting variables
- Statistical models

### Outcomes and prioritisation

The outcomes will be prioritised, i.e. they will be grouped into primary and secondary outcomes, based on the findings from the telephone interviews prior to conducting the review (see the “Consumer involvement” section).

Outcomes may include, but are not limited to the following patient-relevant endpoints:

1. Stoma-related complications:

- Parastomal abscess
- Mucocutaneous separation
- Dermatological complications including peristomal dermatitis / erythema / ulceration
- Stoma ischemia/ necrosis
- Parastomal hernia
- Prolapse
- Stoma stenosis
- Stoma retraction

2. Other patient-relevant postoperative endpoints:

- Revision rate (to manage stoma-related complications)
- Dependence on professional ostomy care
- Mortality (hospital mortality, 30-day mortality, 90-day mortality)
- Length of stay
- Readmission
- Health-related quality of life (measured with a validated tool)
- Anxiety

Any other outcomes not listed above, e.g. because they were detected through the telephone interviews, may also be included. Unless otherwise stated, we will use the timing as defined by the primary study authors.

### Risk of bias in individual studies

We will use the Cochrane ROBINS-I tool (Risk Of Bias In Non-randomized Studies - of Interventions) to assess the risk of bias of observational studies on outcome level [33]. ROBINS-I evaluates baseline and time-varying confounding, co-interventions, selection bias, classification bias, missing data and bias in outcome measurement. If any RCTs or CRCTs will be identified, risk of bias will be evaluated using the Cochrane risk of bias tool 2.0 (RoB 2) on outcome level [34]. The tool evaluates the risk of bias related to the randomisation process, deviations from intended interventions, missing outcome data, measurement of the outcome and selection of the reported result. Based on this, a summary score (overall bias) is created.

Two reviewers will independently assess the risk of bias of the included studies and resolve any disagreements through discussion. In case of insolvable disagreement, a third reviewer (senior researcher) will be involved for arbitration. We will incorporate our risk of bias assessment in the evaluation of the certainty of the body of the evidence [35].

### Data synthesis

We will investigate if there is clinical and/or methodological heterogeneity among the studies. Clinical heterogeneity will be judged based on the clinical insight of the team. Methodological heterogeneity will be investigated by examining all included studies along their characteristics using our methodological judgment.

If the studies are sufficiently homogenous and results’ data are reported adequately, we will perform a meta-analysis to quantitatively pool the studies’ results using the “Review Manager” software (version 5.3) [36]. If not, we will narratively synthesise the studies’ results using tables and/or figures.

In case of meta-analysis, random effects models will be applied as it is unlikely that the studies’ results were identical in their magnitude and direction. We will use the following summary measures: For continuous data, (standardised) mean differences and 95% confidence intervals will be calculated using the inverse-variance method and, for dichotomous data, odds ratios and 95% confidence intervals using the Mantel-Haenszel method [37]. In two sensitivity analyses, we will (1) exclude studies with a high risk of bias and (2) exclude studies which include urostoma patients (studies with < 20% urostoma patients), to assess their influence on the results. Given that the required data are available, we will additionally perform subgroup analyses for all outcomes regarding the:

- Type of hospital (e.g. teaching vs. general)
- Surgeon specialisation (colorectal vs. general)
- Stoma indication (e.g. cancer vs. other diseases)
- Type of stoma (e.g. ileostomy vs. colostomy, temporary vs. permanent)
- Type of person (nurse, medical doctor etc.) performing preoperative stoma site marking

Heterogeneity will be assessed by the Q test and *I*^2^ statistic [38].

### Meta bias(es)

We will assess publication bias by visual inspection of the funnel plots for asymmetry, if the meta-analysis includes at least 10 studies [39]. Furthermore, we will apply Egger’s test and Begg’s test if the number of studies is sufficiently large [40, 41]. A *p* value < 0.1 will be considered statistically significant.

### Confidence in cumulative evidence

We will evaluate the certainty of the body of the evidence using the Grading of Recommendation, Assessment, Development and Evaluation (GRADE) approach [42]. It uses five considerations (study limitations, consistency of effect, imprecision, indirectness and publication bias) to assess the quality of the body of evidence for specific outcomes. Evidence can be downgraded depending on assessments for risk of bias, indirectness of evidence, serious inconsistency, imprecision of effect estimates or potential publication bias [35]. Two reviewers will independently perform GRADE assessment using the GRADEpro GDT software [43]. Summary of finding tables will be prepared for summarising confidence across studies.

### Consumer involvement in outcome prioritisation

Patients should be actively involved in the process of stoma site marking (shared decision-making) since this has a direct influence on ostomy acceptance and quality of life [24].

Public involvement in our SR is particularly important to add value to patients [44]. We will involve patients’ needs, goals, concerns and preferences in our SR to maximise its relevance for clinical practice [44, 45]. To that end, we will conduct semi-structured telephone interviews with patients and ostomy and wound nurses to determine:

- Which outcomes are most important to patients and
- Which non-medical adverse events ostomates have experienced following their ostomy surgery (e.g. problems with stoma care, feeling uncomfortable when performing certain activities, financial burden associated with the acquisition of stoma products).

This information will be used to define and prioritise the endpoints of this SR.

We will develop the two interview guides for patients and ostomy/ wound nurses based on information given by two German ostomate organisations regarding living with a stoma [46] and preoperative marking [47]. In addition, we will discuss questions with the colorectal surgeon of our team concerning preoperative clinical processes. Furthermore, we will get in contact with the ILCO [48] to involve a patient representative with knowledge on ostomy care to critically review the telephone interview guide previously to the pilot interview.

We will pilot each interview guide with one participant. Afterwards, we will include five patients who are heterogeneous in terms of age, gender, socioeconomic status and the ostomy surgery they have undergone as participants. They will be recruited through the German ILCO, which is a solidarity community of ostomates and people with intestinal cancer in Germany as well as their relatives [48] or in the hospital of the clinician of our team. Furthermore, we will include five ostomy and wound nurses as participants. They will be recruited through word of mouth by the clinician of our team.

The semi-structured interviews will be conducted by a researcher with expertise in qualitative research via telephone. We chose this method of data collection because ostomates might feel embarrassed when talking about adverse events, such as stoma leakage. In telephone interviews, participants are generally more likely to disclose sensitive information and feel comfortable than during face-to-face interviews/focus groups [49]. Additionally, this method is in line with contact restrictions during the Covid19 pandemic in 2020. All interviews will be recorded and transcribed verbatim for qualitative content analysis according to Mayring [50]. For coding, we will use the open access, interactive, internet-based program QCAmap [51]. Participants will be informed about the aim of the study, study process and data protection. We will conduct written informed consent from each participant prior to the interviews. Each participant will receive 125€ for their participation.

We will conduct the semi-structured telephone interviews before initiating the SR. The patient representative will be invited to discuss the manuscript for the SR protocol and the manuscript for the completed SR during meetings [45]. He or she will be involved continuously over the course of the SR.

### Plan for documenting important protocol amendments

Important protocol amendments will be documented in PROSPERO as well as in the review publication.

## Discussion

With the results of the SR, we aim to provide information for future clinical guidelines and influence clinical routine with regard to the practice of preoperative stoma site marking in patients undergoing ostomy surgery. This in turn may reduce the burden of disease due to stoma-related complications and increase the quality of life of ostomates. It could also have a positive economic impact on the healthcare system due to less treatment costs for the management of stoma-related complications.

When the quality of the evidence of our SR is low, it will remain unclear if preoperative stoma site marking may indeed be effective in reducing or preventing stoma-related complications. This being the case, it will still be a useful basis for future clinical trials. It will include a detailed description of the characteristics of existing studies and identify evidence gaps.

## Supplementary Information

**Additional file 1.** PRISMA-P Checklist.

## Data Availability

The datasets generated and/or analysed during the current study will be made available in the Systematic Review Data Repository (SRDR+), https://srdr.ahrq.gov/

## Abbreviations

GRADE: Grading of Recommendation, Assessment, Development and Evaluation
RCT: Randomised controlled trial
ROBINS-I: Risk Of Bias In Non-randomized Studies - of Interventions
SR: Systematic review
SRDR+: Systematic Review Data Repository
